# Role of the alternative splice variant of NCC in blood pressure control

**DOI:** 10.1080/19336950.2018.1528820

**Published:** 2018-09-28

**Authors:** Hila Wardak, Omar A.Z. Tutakhel, Jenny Van Der Wijst

**Affiliations:** a Department of Physiology, Radboud Institute for Molecular Life Sciences, Radboud university medical center, Nijmegen, The Netherland; b Department of Translational Metabolic Laboratory, Radboud university medical center, Nijmegen, The Netherlands

**Keywords:** Kidney, NCC, signaling, splice variant, urinary extracellular vesicles

## Abstract

The renal thiazide-sensitive sodium-chloride cotransporter (NCC), located in the distal convoluted tubule (DCT) of the kidney, plays an important role in blood pressure regulation by fine-tuning sodium excretion. The human *SLC12A3* gene, encoding NCC, gives rise to three isoforms, of which only the third isoform (NCC_3_) has been extensively investigated so far. However, recent studies unraveled the importance of the isoforms 1 and 2, collectively referred to as NCC splice variant (NCC_SV_), in several (patho)physiological conditions. In the human kidney, NCC_SV_ localizes to the apical membrane of the DCT and could constitute a functional route for renal sodium-chloride reabsorption. Analysis of urinary extracellular vesicles (uEVs), a non-invasive method for measuring renal responses, demonstrated that NCC_SV_ abundance changes in response to acute water loading and correlates with patients’ thiazide responsiveness. Furthermore, a novel phosphorylation site at serine 811 (S811), exclusively present in NCC_SV_, was shown to play an instrumental role in NCC_SV_ as well as NCC_3_ function. This review aims to summarize these new insights of NCC_SV_ function in humans that broadens the understanding on NCC regulation in blood pressure control.

## Introduction

Hypertension (high blood pressure) is a major risk factor for stroke, myocardial infarction, heart failure, chronic kidney disease and overall increased risk of mortality [1–4]. It is the most critical and expensive public health problem with a global prevalence of 1.4 billion people, representing one of every four adults worldwide. This is predicted to increase to 1.6 billion people by 2025 [1,2,5–7]. About 90% of hypertensive patients have primary or essential hypertension, which means that the origin of their disorder is unknown and secondary causes such as monogenic disease, renal failure, aldosteronism and renovascular disease are not present [8,9].

It is well known that salt intake plays a significant role in the development of hypertension. The body’s salt homeostasis is maintained by the kidneys through regulated processes of sodium (and chloride) reabsorption [10]. Specifically, the sodium-chloride cotransporter (NCC) is an important player in the maintenance of salt homeostasis as it controls the fine-tuning (~ 7%) of sodium excretion in the distal convoluted tubule (DCT) [11]. NCC is an important pharmacological target in the treatment of hypertension as thiazide-type diuretics, which specifically block NCC, are considered first-line therapy [12,13]. Furthermore, over 180 loss-of-function mutations have been identified in the *SLC12A3* gene, encoding NCC, in relation to Gitelman syndrome (OMIM 263,800) [14–16]. This is an autosomal recessive disease that is portrayed by hypocalciuria, hypomagnesemia, hypokalemic metabolic alkalosis, sodium wasting and lower blood pressure levels in comparison to their age-matched unaffected relatives [17,18].

The human *SLC12A3* gene yields three separate isoforms as a result of an alternative splicing. NCC isoforms 1 and 2 (NCC_1_ and NCC_2_), collectively termed NCC_SV_, are nine amino acids longer than the third isoform (NCC_3_) [18–20]. In the present review, we aim to outline the newest findings on the molecular regulation of NCC_SV_ and its role in renal salt handling in various (patho)physiological conditions. A better understanding of all three isoforms is crucial to unravel the molecular events underlying the pathogenesis of essential hypertension, and to develop effective therapeutic approaches to combat the dysregulation of blood pressure.

## Characterization of NCC_SV_

**Figure 1.**
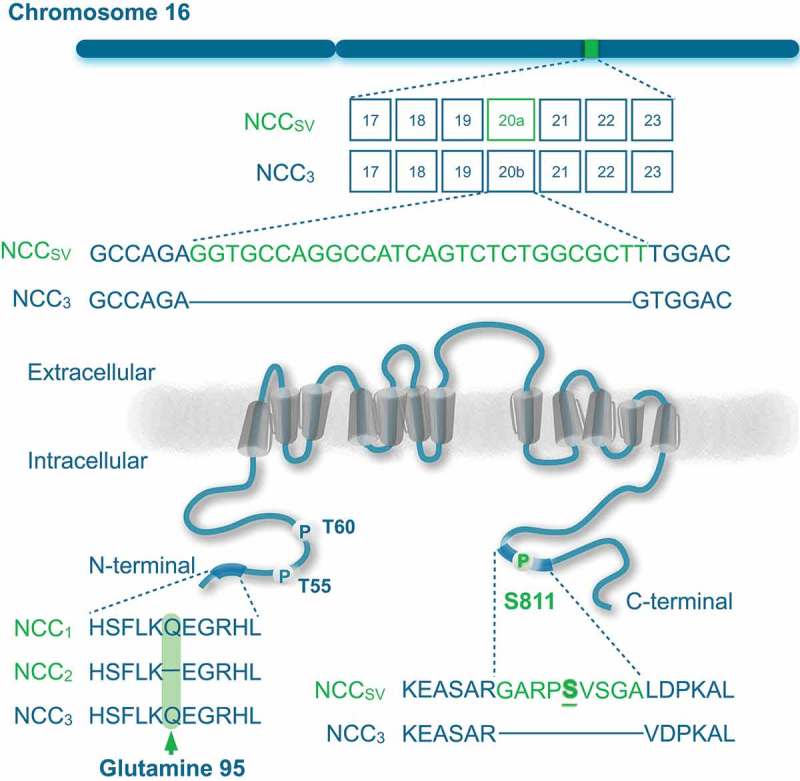
Splicing of NCC gene. Schematic representation of the *SLC12A3* gene including exon 20a that encodes for the NCC_SV_ (top). The encoded protein NCC contains 12 transmembrane domains and intracellular amino (N)- and carboxy (C)-terminal domains (bottom). Serine (S811) is highlighted as phosphorylation site in the nine additional amino acids in NCC_SV_. Left bottom panel shows a multiple protein sequence alignment of the three NCC isoforms, demonstrating the lack of glutamine 95 in NCC_2_.

The molecular cloning of human NCC took place in 1996, showing that there exist three different isoforms of the protein as a result of alternative splicing of the *SLC12A3* gene [18,21]. Simon *et al*. reported an mRNA sequence encoding a 1,030 amino acid protein, which was NCC_1_ [18]. Mastroianni *et al*. reported later the mRNA sequence corresponding to the NCC_3_, which encoded for a protein of 1,021 amino acids [21]. The exon 20 of NCC_3_ is nine amino acids shorter in comparison to NCC_1_. A possible cryptic splicing at the donor site leads to both long and short forms of exon 20 [19]. In comparison to NCC_1_, the isoform 2 (NCC_2_) lacks one amino acid at glutamine residue 95 within amino (N)-terminal domain, making these two isoforms practically indistinguishable (Figure 1). Hence, both isoforms are collectively referred to as NCC splice variant (NCC_SV_), which is only present in humans and higher primates.

Recently, the presence of both NCC_3_ and NCC_SV_ was shown in human urinary extracellular vesicles (uEVs) using mass spectrometry and immunoblot analysis [20]. This study confirmed multiple overlapping peptides of NCC_SV_ and NCC_3_, which revealed that *SCL12A3* leads to the expression of a mixture of NCC_3_ and at least one but possibly both NCC_1_ and NCC_2_ [20]. Total NCC mRNA in the human kidneys consisted on average of ~ 45% of NCC_SV_ and ~ 50% of NCC_3_ [20]. However, the ratio of NCC_SV_ to NCC_3_ mRNA expression in human kidney varied between 20 and 60%, which is likely the result of a highly regulated splicing at exon 20 [20]. It has been shown that ~ 95% of human genes are alternatively spliced and the production of alternative mRNA splices is diversely regulated by a variety of external factors. Mechanisms of alternative splicing are highly variable and not well understood yet [22].

Generation of antibodies specific against NCC_SV_ led to confirmation of NCC_SV_ abundance at the apical membrane of the DCT in human kidney, as well in kidney membrane fractions and uEVs [20]. This demonstrated the potential importance of the formerly underrepresented NCC_SV_ in renal salt handling.

## Functional role of NCC_SV_

### Regulators of NCC

NCC is a member of the solute carrier 12 (SLC12) family of electroneutral cation-coupled chloride cotransporters. It is a membrane glycoprotein and contains 12 transmembrane segments (S) with large intracellular N- and carboxy (C)-terminal regions, and a large hydrophilic extracellular loop between S7 and S8 [23]. This loop contains glycosylation sites that are essential for the cell surface expression, thiazide sensitivity and activity of NCC [24]. Functional NCC proteins represent as homodimer at the plasma membrane [25,26]. Other posttranslational modifications that play a crucial role in the regulation of NCC activity include ubiquitylation and phosphorylation (reviewed in [27–29]. According to recent large-scale proteomics studies, NCC is ubiquitylated on several conserved lysine residues [30,31]. Generally, NCC ubiquitylation is associated with increased endocytosis from the plasma membrane, as well as protein degradation [32,33]. A recent study pointed out that NCC exhibits site-specific ubiquitylation exerting different roles on NCC function, via modulating NCC phosphorylation or its total abundance at the plasma membrane [33]. Interestingly, several studies indicate that phosphorylation of NCC can interfere with its ubiquitylation, thereby playing a dual role in modulating NCC function [34,35]. Generally, phosphorylation is considered one of the most important posttranslational modification processes in the regulation of NCC activity. The N-terminal domain of NCC contains several key phosphorylation sites including threonines 48, 55 and 60 (T48, T55, T60) and serines 73, 91 and 124 (S73, S90, S124) [36–38]. Phosphorylation at these sites is important for plasma membrane abundance and influence NCC activity [29,39]. Interestingly, several studies have shown that changing T60 to a non-phosphorylated amino acid (T60A) prevented or reduced the phosphorylation at other sites (T48, T55, S73 or S91), suggesting that T60 is a key modulator in the regulation of NCC activity [29,35,37]. This is highlighted by a loss-of-function mutation at T60 in NCC that is linked to Gitelman Syndrome [35,40].

**Figure 2.**
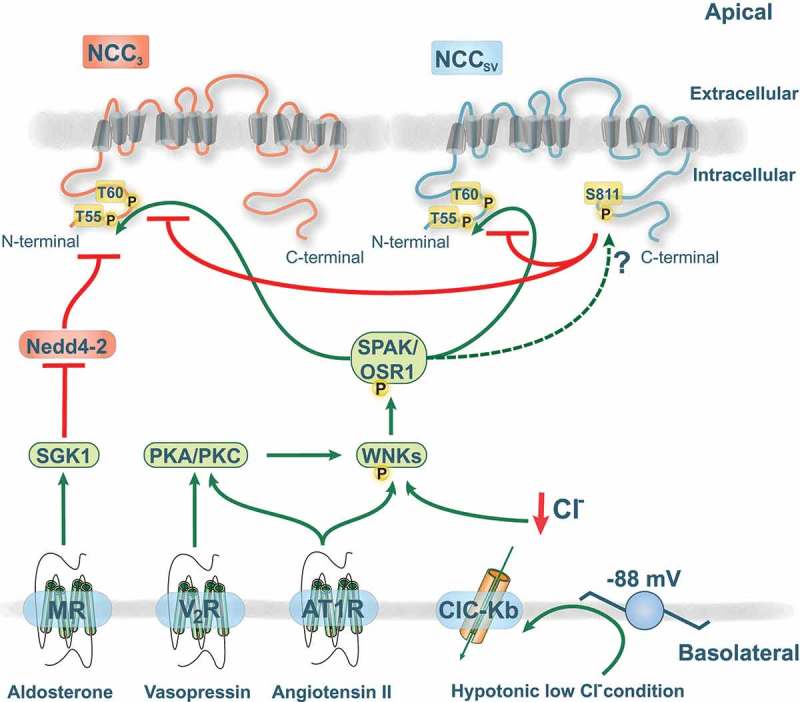
Functional role of the novel phosphorylation site serine 811 (S811). The amino (N)-terminal domain of NCC contains several key phosphorylation sites (T55/T66) that are essential for NCC activity and plasma membrane abundance. S811 acts as a dominant regulatory site for phosphorylation of T60 and T55 in NCC_SV_ and NCC_3_. The various interactions of the NCC regulatory pathway are shown as green arrows (stimulatory) or red lines (inhibitory). Phosphorylation is indicated with the symbol P. The with no lysine kinases (WNKs) are capable of activating SPAK (STE20/SPS1-related, proline alanine-rich kinase) and OSR1 (oxidative stress responsive protein type 1). The hormone receptors are located in the basolateral membrane with indicated signaling pathways involving either serum glucocorticoid regulated kinase 1 (SGK1)-Nedd4-2 pathway, protein kinase A/C (PKA/PKC), or WNKs. V2R, vasopressin receptor; AT1R, angiotensin receptor 1; MR, mineralocorticoid receptor.

These phosphorylation sites (apart from S124) are targets of the serine-threonine kinases SPAK (STE20/SPS1-related, proline alanine-rich kinase) and OSR1 (oxidative stress responsive protein type 1), which are activated by with no lysine (WNK) kinases [37,41]. Mutations in the human genes encoding WNK1 and WNK4 are causing Gordon’s syndrome [42], which is characterized by hypertension, hyperkalemia, metabolic acidosis, and hypercalciuria. This phenotype is the mirror image of Gitelman syndrome, and it was shown to result from gain-of-function of NCC [43]. While the exact mode of action of the different WNK kinases is still under debate, there are three abundant WNK kinases along the DCT, namely WNK1, WNK3, and WNK4, that affect NCC function by modulating trafficking and/or transport activity via phosphorylation [44]. Regulation of NCC by the WNK-SPAK signaling is an area of intense research and several studies also provided evidence for hormone regulation of the WNK-SPAK-NCC pathway (Figure 2) reviewed in [45] and [29].

### Novel phosphorylation site in NCC_SV_

All three human NCC isoforms contain the T55 and T60 residues that are essential mediators of NCC activity. Recently, a new phosphorylation site uniquely located at S811 in NCC_SV_ was identified [46]. Tutakhel *et al*. further investigated the role of NCC_SV_ S811 phosphorylation on activity of both NCC_SV_ and NCC_3_ [47]. They showed that a non-phospho-mimetic (S811A) mutant of NCC_SV_, but not the phospho-mimetic S811D, prevents the phosphorylation at T55 and T60. Importantly, NCC_SV_ S811A was able to inhibit NCC_3_ phosphorylation at T55 and T60 upon co-expression of NCC_SV_ and NCC_3_ [47]. In both phosphorylation sites the decrease was more than 50%. This indicates that S811 not only regulates NCC_SV_ activity, but also affects the function of NCC_3_ in a dominant-negative fashion (Figure 2). This effect may be due to the heterodimerization of NCC_SV_ with NCC_3_. The translocation of NCC to the plasma membrane or stability of NCC did not seem to be affected by S811 [47]. This is in agreement with a study demonstrating that NCC_3_ surface abundance is not affected by the phosphorylation status of T60 [35,36,48]. Based on these insights, NCC_SV_ might have a crucial role in renal salt handling.

Although it is acknowledged that T60 and T55 are phosphorylated via activation of the WNK-SPAK signaling pathway [36,49], the kinase involved in S811 phosphorylation remains to be clarified (Figure 2). Prediction software (pkaPS) from the IMP-IMBA Bioinformatics group [50] suggested that the recognition motif around S811 of NCC_SV_ shows high similarity to a protein kinase A (PKA) consensus site. Interestingly, several hormonal systems have been shown to activate the cAMP-PKA pathway [51,52]. For example, the sodium-potassium-chloride cotransporter (NKCC2), a SLC family member in the thick ascending limb of Henle’s loop (TAL), can be phosphorylated by PKA signaling downstream of hormones like arginine vasopressin (AVP) or parathyroid hormone (PTH) [53,54]. Additionally, an increasing amount of evidence suggests that the abundance and phosphorylation of NCC is affected by AVP and angiotensin II, which might include PKA or protein kinase C (PKC) [55–58] (Figure 2). While there is no information yet on direct effects on NCC, it was recently shown that activation of PKC by angiotensin II prevents the binding of WNK4 by kelch-like 3 (KLHL3). KLHL3, together with the ubiquitin ligase cullin 3 (CUL3), normally targets WNK4 for ubiquitylation and degradation [59]. PKC can phosphorylate S433 in KLHL3, a site that was previously postulated to be targeted by PKA as well [60]. In addition, PKC was shown to phosphorylate WNK4 directly, leading to an increase in kinase activity [55]. Hence, PKA and PKC activation can stimulate WNK4 signaling, indirectly leading to NCC activation. The NCC cell surface abundance and phosphorylation are also increased by the mineralocorticoid aldosterone, likely through a mechanism involving the serum glucocorticoid regulated kinase 1 (SGK1)-Nedd4-2 pathway or WNK4 [45,61] (Figure 2). However, recent evidence suggests that modulation of NCC in response to aldosterone is mediated by secondary changes in plasma potassium concentration that result from aldosterone effects on ENaC [62,63]. Interestingly, while most of the studies on NCC regulation have been performed in animals, this point was recently corroborated in humans [64]. Mineralocorticoid administration in humans resulted in increased NCC and WNK4 abundance, as well as enhanced NCC phosphorylation. This was negatively correlated with plasma potassium levels, supporting the role of potassium effects on NCC, possibly via WNKs [64].

Altogether, NCC regulation in response to the renin-angiotensin-aldosterone (RAAS) system and AVP has been studied extensively and is well documented [45]. It will be interesting to study whether these hormones also signal towards NCC_SV_ and identify which pathway is involved in S811 phosphorylation.

## The role of NCC_SV_ in (patho)physiology

### Urinary extracellular vesicles, a tool to study NCC

Over the past years, uEVs have been established as a non-invasive tool to study NCC regulation in several physiological and pathophysiological conditions. The uEVs are composed of nucleic acids and proteins that reflect the (patho)physiological state of cells lining the nephron and the urinary tract. They contain membrane and cytosolic proteins, DNA and RNA that are conserved and protected from degradation [65,66]. The cargo of uEVs reflects changes in the expression of different proteins present in the epithelial cells of the nephron, including the total and phosphorylated forms of NCC [20,67,68]. Indeed, it has been demonstrated that patients with Gitelman syndrome display a decreased NCC abundance in uEVs [67,69]. On the other hand, patients with Gordon syndrome show higher levels of NCC in the kidney and this increased abundance was also observed in the uEVs [68]. Moreover, a recent study on kidney transplant recipients treated with calcineurin inhibiters (CNIs) suggested the use of NCC abundance in uEVs as biomarker to predict hypertensive patient’s response to thiazide diuretics [70]. The CNIs cyclosporine A and tacrolimus are commonly used to prevent rejection of transplanted organs, and a common side effect of these drugs is hypertension that can be accompanied by hyperkalemia and metabolic acidosis [71]. Recent studies showed increased levels of total and phosphorylated NCC in uEVs of patients with chronic CNI treatment [70], which suggests that NCC might be involved in the pathogenesis of hypertension in kidney transplant recipients. Hence, the NCC abundance in uEV was analyzed in these patients following treatment with thiazide diuretics, the main anti-hypertensive drug. In line with several studies in animal models [72,73], NCC abundance was increased after thiazide treatment [70]. Furthermore, pre-treatment abundance of NCC in uEVs correlated with blood pressure response to thiazide diuretics, which implies that this method could be used to predict patients’ thiazide sensitivity. Upon dividing the group in responders (blood pressure decrease ≥ 10 mmHg) and non-responders, it became apparent that the non-responder group had a decrease in the ratio between phosphorylated and total NCC (pNCC/tNCC) [70]. Moreover, the serum potassium concentration was lower in responders compared to non-responders, which might be explained by the fact that blood pressure response to thiazides is accompanied by altered renal potassium handling. Blocking of NCC likely results in increased distal delivery of sodium that results in increased potassium secretion via the renal outer medullary potassium channel (ROMK).

### NCC_SV_ in physiology

Both NCC_SV_ and NCC_3_ are present as glycosylated oligomeric structures in uEVs [74,75]. Hence, uEVs were used to assessed the role of NCC_SV_ *in vivo*. In response to water loading, a strong decrease in expression of NCC_3_ as well as NCC_SV_ was shown [20]. Water loading results in transient extracellular volume expansion and decrease in serum osmolality, which is known to deactivate RAAS and reduce AVP release [76]. AVP is mainly known for its water-retaining action on collecting duct (CD) by increasing the abundance of the water channel aquaporin 2 (AQP2) [77]. Two recent studies demonstrated that water loading indeed resulted in significantly reduced AQP2 expression in uEVs of the healthy volunteers [20,78]. In line with this, urine osmolality was also decreased, reflecting the diluted urine after water loading [20,78]. Since several studies have described the regulation of NCC by angiotensin, aldosterone, and AVP (reviewed in [45]), the decrease in NCC_SV_ abundance in water-loaded subjects is likely the result of the decline in RAAS activity and AVP release [20]. So far, it indicates that NCC_SV_, like NCC_3_, is highly regulated under physiological conditions and suggests a key role in renal salt handling. As abovementioned, future studies should examine whether AVP or the RAAS hormones are directly involved in phosphorylation of NCC_SV_.

### NCC_SV_ in pathophysiology

To understand the role of NCC_SV_ in pathophysiological conditions, the abundance and phosphorylation of all three NCC isoforms were compared in uEVs of essential hypertensive patients before and after hydrochlorothiazide or valsartan treatment [75]. The thiazide-like diuretics work as an anti-hypertensive medication by direct blocking of NCC [79], whereas valsartan is a well-known anti-hypertensive drug that reduces blood pressure by antagonizing the angiotensin II type 1 receptor [80].

While plasma sodium levels remained the same in both treatments, plasma renin levels increased considerably after valsartan treatment. Plasma potassium concentrations were reduced in patients treated with hydrochlorothiazide but not upon valsartan treatment [75]. Interestingly, the abundance of NCC_SV_ and NCC_3_ was increased upon chronic thiazide treatment, but not by valsartan [75]. However, dividing the hydrochlorothiazide patient group into responders (blood pressure decrease ≥ 5 mmHg) and non-responders revealed that the NCC_SV_ and NCC_3_ abundance was significantly higher in responders than in patients who did not respond to thiazide treatment. There was no significant difference of the phosphorylated form of NCC (T55/T60 abundance) [75]. These data highlighted that the blood pressure decrease upon thiazide treatment correlates with NCC_SV_ abundance in uEVs and with plasma potassium levels. Together, these observations demonstrate the importance of NCC_SV_ in pathophysiological processes and its involvement in blood pressure control.

## Future perspectives

NCC_SV_ is significantly expressed in the human kidney and is not a redundant transcription product, but a fully functional thiazide-sensitive sodium-chloride transporting protein. Besides, the phosphorylation site S811 in NCC_SV_ acts as a dominant-negative regulatory site for phosphorylation of T55 and T60 in all isoforms. While the WNK-SPAK kinases are a well-studied signaling mechanism for NCC regulation, it is yet unclear whether which molecular pathways and kinases are involved in phosphorylation of S811. Future experiments should focus on delineating the regulation of NCC_SV._ These discoveries reveal a new regulatory mechanism of NCC function, which might be important in renal salt handling, consequently playing a significant role in the pathogenesis of essential hypertension. Future experiments developing S811 phospho-specific antibodies and samples from different human pathological states could provide further understanding of the precise role of NCC_SV._ Here, uEVs can serve as a non-invasive biomarker source as recent data revealed its potential in guiding anti-hypertensive therapy in patients with essential hypertension. This could ultimately lead to personalized anti-hypertensive treatment. The development and standardization of high-throughput assays for uEV analysis will be necessary for future clinical applications.
